# Supplementary material for: The release of toxic oligomers from α-synuclein fibrils induces dysfunction in neuronal cells
Source: Nat Commun. 2021 Mar 22;12:1814. doi: 10.1038/s41467-021-21937-3 (PMC7985515; doi:10.1038/s41467-021-21937-3)
Supplement: Supplementary file 1 — Supplementary Information [file 41467_2021_21937_MOESM1_ESM.pdf]

SUPPLEMENTARY TABLES

Supplementary Table 1 – Morphological and structural properties of the different αS species reported in Fig. S1.

	Height (nm)	Length (nm)	β-sheet content (%)	cross-β signature	ThT binding (amyloid structure)	ANS binding (solvent-exposed hydrophobic surface)
M	1.2±0.3	-	0	NO	NO	NO
OA*	5.1±0.8	28±6	0	NO	NO	NO
OB*	4.3±0.9	32±5	30	YES	Low affinity/signal	High affinity/signal
SF	5.0±2.6	57	65	YES	High affinity/signal	High affinity/signal
LF	6.2±3.8	520	65	YES	High affinity/signal	High affinity/signal

SUPPLEMENTARY FIGURES AND LEGENDS

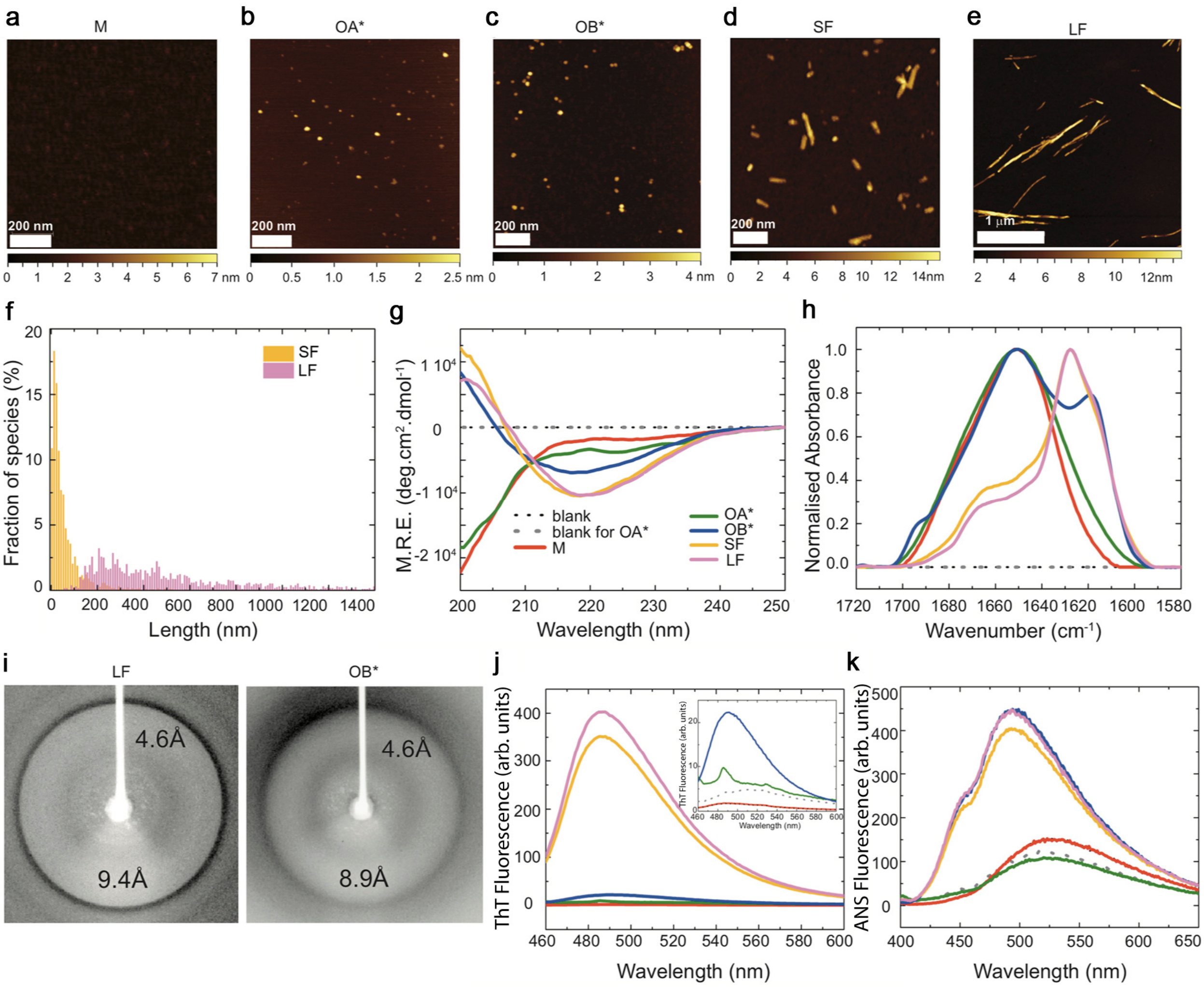

Supplementary Figure 1. Morphological and structural characterization of the different αS species. (a-e) AFM images (height data) of the various αS species: M/OA*/OB*/SF/LF (three independent experiments with one internal replicate). The white and coloured bars represent the length and height scales, respectively. Both OA* and OB* are globular and apparently spherical with similar average heights, 5.1±0.8 nm (n=300) and 4.3±0.9 nm (n=300), respectively; SF and LF adopt elongated morphologies with averaged heights of 5.0±2.6 nm (n=300) and 6.2±3.8 nm (n=300), respectively. (**f**) The length distribution of the different fibril population as estimated by AFM. SF and LF show average lengths of 57 nm (n=2035) and 520 nm (n=1415), respectively. **(g-h)** Far-UV CD and FT-IR spectra of the five αS species. OA* show predominantly disordered secondary structure with similar spectra to those of M, while OB*/SF/LF possess a β-sheet core with an average of ~ 30-35% of the protein sequence in a β-sheet conformation for OB*, and ~ 65% for the fibrils. (**i**) X-ray diffraction patterns of OB* and LF. OB* display a cross-β structure with an inter-strand spacing of 4.6 Å and an inter-sheet distance of 8.9 Å, closely similar to the values of 4.6 and 9.4 Å, respectively, exhibited by the LF. **(j)** ThT fluorescence spectra of the five αS species. The cross-β structure of OB* is rudimentary and show deficiencies in regularity and compactness as compared to the fibrils. The inset is the same plot on an expanded *y* scale. **(k)** ANS fluorescence spectra of the five αS species. OB*/SF/LF show a virtually identical blue-shift of the wavelength of maximum emission, indicating a similar and high degree of solvent-exposed hydrophobic surface area. M/OA* have a very low hydrophobic surface exposed to the solvent.

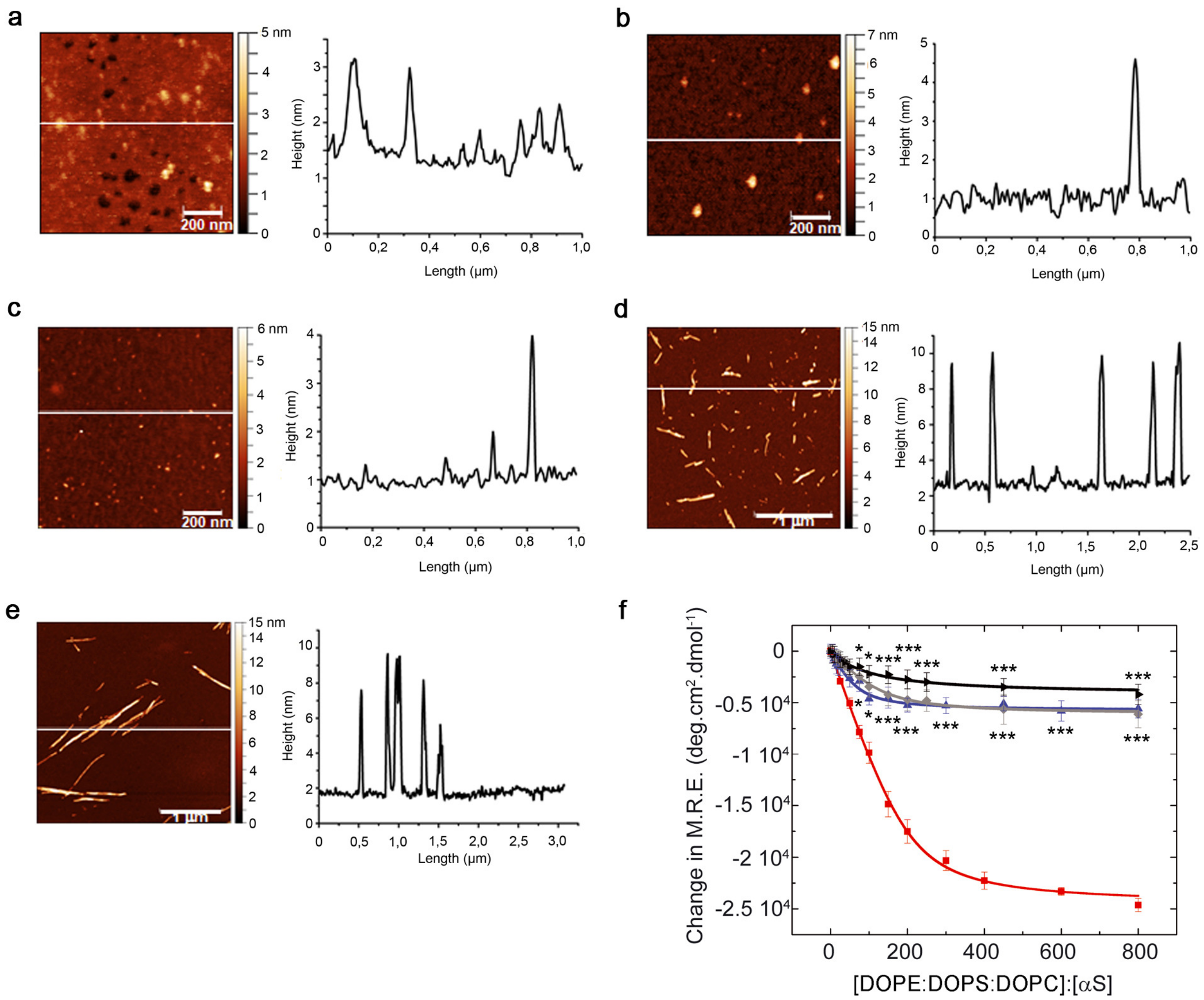

Supplementary Figure 2. AFM analysis of the different αS species and interaction of the different αS cross-β aggregates with lipid vesicles. (a-e) Representative AFM images and cross-section analysis derived from experiments described in Supplementary Figure 1 (three independent experiments with one internal replicate) of M **(a)**, OA* **(b)** OB*, **(c)** SF **(d)** and LF **(e)** samples are shown for comparison. (**f**) Far-UV mean residue ellipticity (222 nm) induced in αS upon binding to SUVs containing DOPE:DOPS:DOPC with a molar ratio of 5:3:2 (acquisition of helical structure upon binding). The data is reported as change in mean residue ellipticity (M.R.E.) as a function of the ratio between the concentration of lipids and protein (mass concentration) for each αS species: M in red, OB* in blue, SF in grey and LF in black. In panel f, experimental errors are S.E.M. (n=3 with one internal replicate). Samples were analyzed by one-way ANOVA followed by Bonferroni's multiple comparison test relative to M (* $P<0.05$ and ***$P<0.001$).

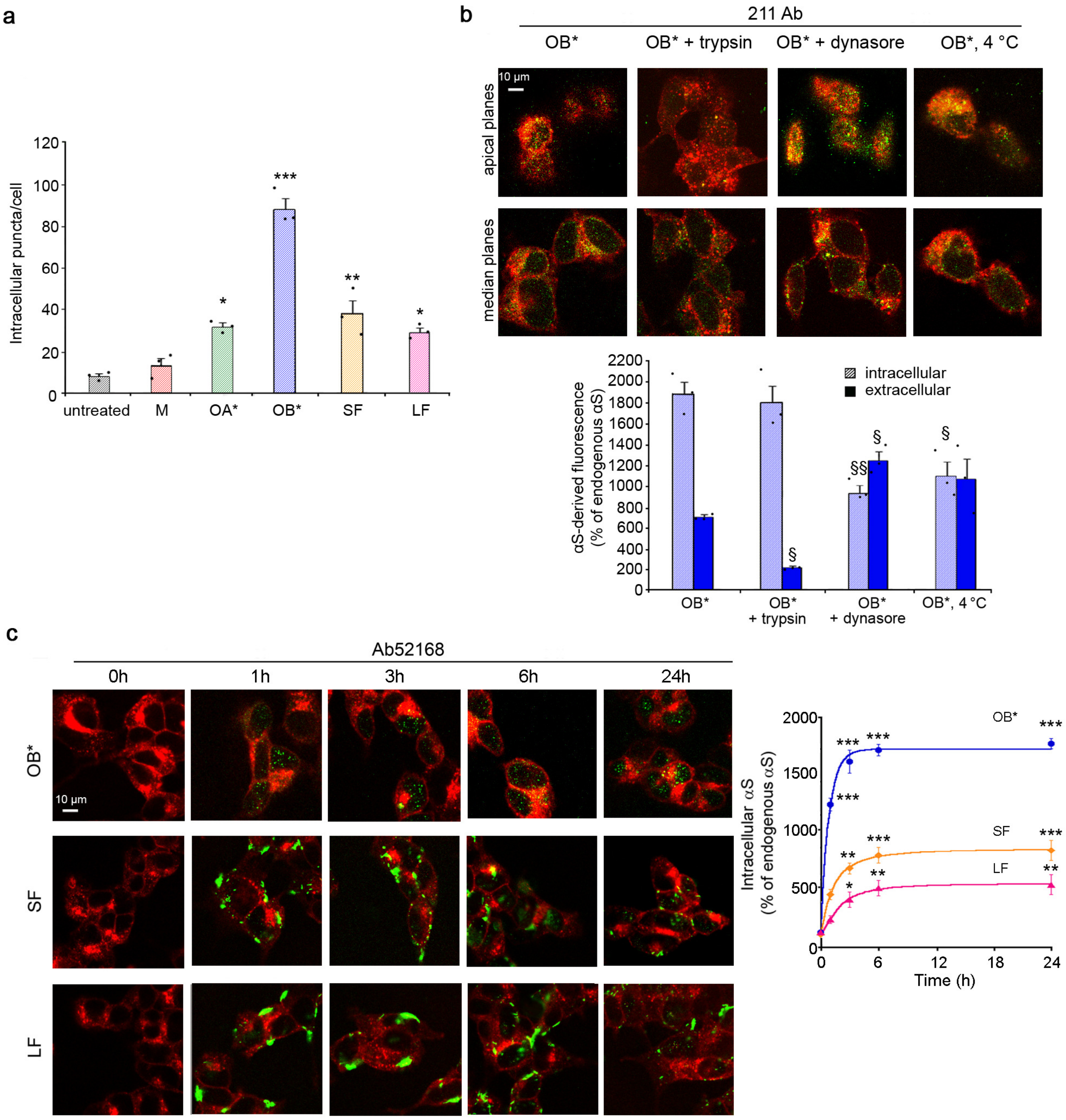

Supplementary Figure 3. Intracellular uptake of different αS species in SH-SY5Y cells following different lengths of time. (a) Semi-quantitative analysis of the intracellular αS-derived fluorescence data showed in Fig. 2a expressed as number of puncta per cell. (**b**) Representative confocal scanning microscope images showing the apical and median sections of SH-SY5Y cells treated for 6 h with 0.3 μM OB* at 37 °C and then kept for 15 min at 4 °C, or for 6 h with 0.3 μM OB* at 37 °C and then with 0.05% trypsin for 15 min at 4 °C, or for 6 h at 37 °C with 0.3 μM OB* in the presence of 5 μM dynasore or for 6 h with 0.3 μM OB* at 4 °C. Red and green fluorescence indicates the cell membranes and the αS species revealed with WGA and mouse monoclonal 211 anti-αS antibodies (sc12767, Santa Cruz Biotechnology), respectively. Semi-quantitative analysis of the green fluorescence signal derived from intracellular and extracellular αS. (**c**) Representative confocal scanning microscope images showing the median sections of SH-SY5Y cells treated for 0, 1, 3, 6 and 24 h with OB*/SF/LF at 0.3 μM concentration. Red and green fluorescence indicates the cell membranes and the αS species revealed with WGA and polyclonal anti-αS antibodies

(ab52168, Abcam), respectively. The kinetic plots report αS-derived intracellular fluorescence following the addition of 0.3 μM of the indicated αS species. The lines represent the best fits to exponential and sigmoidal functions (see Methods), for OB*, SF and LF, respectively. Experimental errors are S.E.M. (n=3 with four internal replicates in panel a; n=3 with one internal replicate in panel b; n=4 with one internal replicate in panel c). In all panels, samples were analyzed by one-way ANOVA followed by Bonferroni's multiple comparison test relative to untreated cells (* $P<0.05$, **$P<0.01$, ***$P<0.001$) or to cells treated with OB* (§$P<0.05$, §§$P<0.01$). A total of 200–250 cells were analyzed per condition.

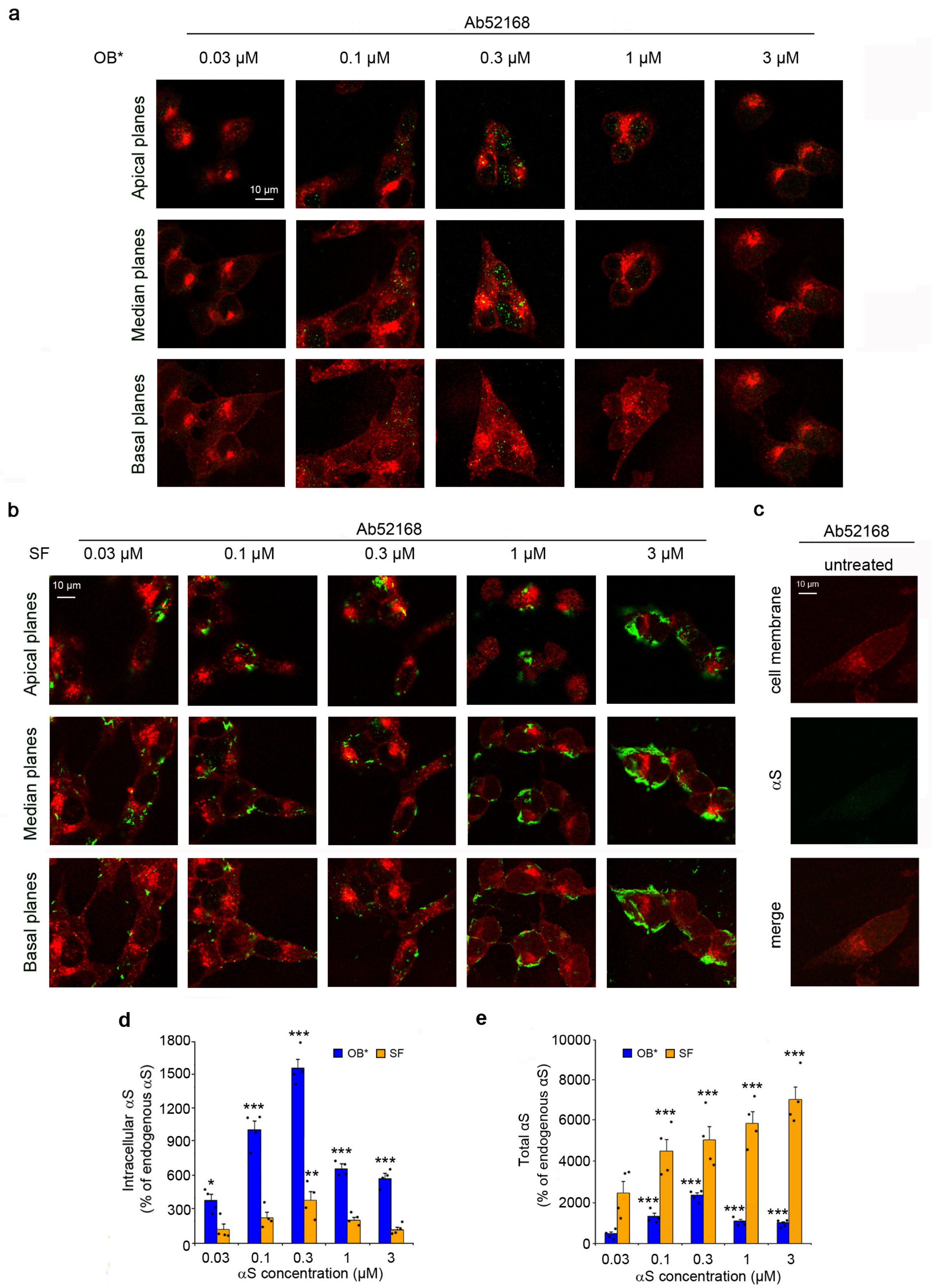

Supplementary Figure 4. Intracellular uptake of different αS species in cells. (a-b) Representative confocal scanning microscope images showing the basal, median and apical sections of SH-SY5Y cells treated for 1 h with OB* (**a**) and SF (**b**) at the indicated αS concentrations. Red and green fluorescence indicates the cell membranes and the αS species revealed with WGA and polyclonal anti-αS antibodies (ab52168, Abcam), respectively. (**c**)

Representative confocal scanning microscope images showing the cell membrane (red), the endogenous αS (green) and the merge of all sections of untreated SH-SY5Y cells (four independent experiments with three internal replicates). **(d-e)** Semi-quantitative analysis of the green fluorescence signal derived from intracellular **(d)** and total **(e)** αS. Experimental errors are S.E.M. (n=4 with three internal replicates). In all panels, samples were analyzed by one-way ANOVA followed by Bonferroni's multiple comparison test relative to untreated cells (*P<0.05, **P<0.01, ***P<0.001). A total of 200–250 cells were analyzed per condition.

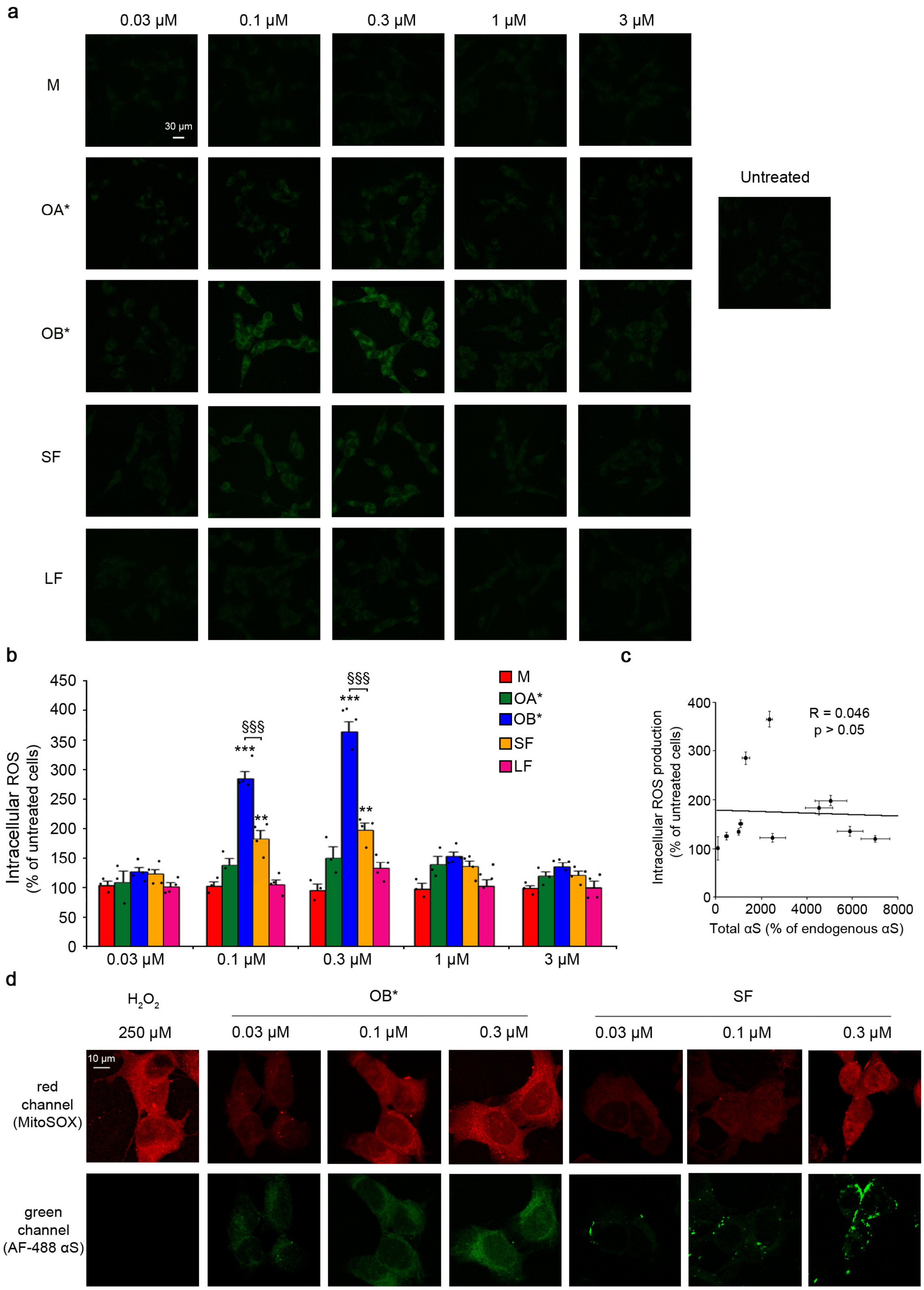

Supplementary Figure 5. ROS production in cells exposed to different αS species. (a) Representative confocal microscope images showing intracellular ROS levels in SH-SY5Y cells treated for 15 min with the indicated αS species at the indicated αS concentrations.

Untreated cells are also shown. The green fluorescence arises from the CM-H_2DCFDA probe that has reacted with ROS. (**b**) Semi-quantitative analysis of the intracellular ROS-derived fluorescence, expressed as the percentage of the value for untreated cells. (**c**) Dependence of ROS production on the total αS-derived fluorescences in cells treated with OB* and SF**.** ROS values reported in panel B were plotted against the αS-derived fluorescences reported in Fig. S4E of SH-SY5Y cells treated with OB* and SF at the corresponding concentrations. (**d**) Representative confocal scanning microscope images showing mitochondrial superoxide production detected with the MitoSOX probe in SH-SY5Y cells treated for 1 h with OB* and SF labeled with AF488 dye. Positive control with 250 µM H_2O_2 is also showed (six independent experiments with one internal replicate). Experimental errors are S.E.M. (In pane b and c n=4 with three internal replicates for OB*, SF and LF, n=3 with two internal replicates for M and OA*). In panels b-c, samples were analyzed by one-way ANOVA followed by Bonferroni's multiple comparison test relative to untreated cells (**P<0.01, ***P<0.001), or to cells treated with OB* (§§§P<0.001). A total of 200–250 cells were analyzed per condition.

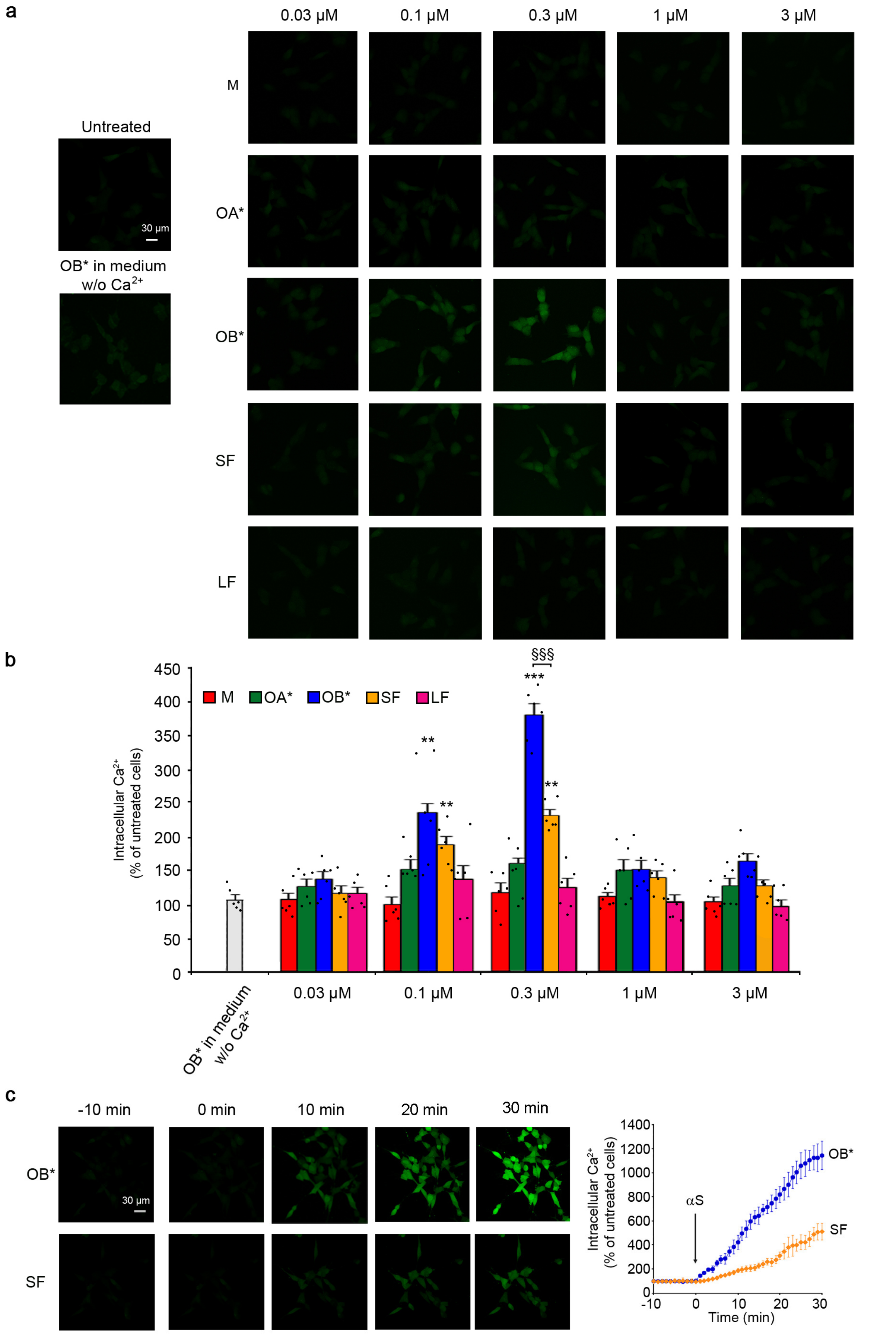

Supplementary Figure 6. Ca^{2+} influx in cells exposed to different αS species. (a) Representative confocal microscope images showing intracellular Ca^{2+} levels in SH-SY5Y

cells treated for 15 min with the indicated αS species at the indicated αS concentrations, untreated cells and cells treated with 0.3 μM OB* in CM without Ca^{2+}. Cells were loaded with Fluo-4AM probe. (**b**) Semi-quantitative analysis of the intracellular Ca^{2+}-derived fluorescence, expressed as the percentage of the value for untreated cells. (**c**) Representative confocal microscope images showing real-time intracellular Ca^{2+} measurement in SH-SY5Y living cells up to 30 min treatment with the indicated αS species at a concentration of 0.3 μM. Kinetic plots reported the fluorescence associated with Fluo-4 AM versus time elapsed after αS species addition to the CM. The continuous lines represent the best fits to sigmoidal functions (see Methods), for OB* and SF. Experimental errors are S.E.M (in panel b and c n=6 with one internal replicate). Samples were analyzed by one-way ANOVA followed by Bonferroni's multiple comparison test relative to untreated cells (**P<0.01, ***P<0.001) , or to cells treated with OB* (§§§P<0.001). A total of 200–250 cells were analyzed per condition.

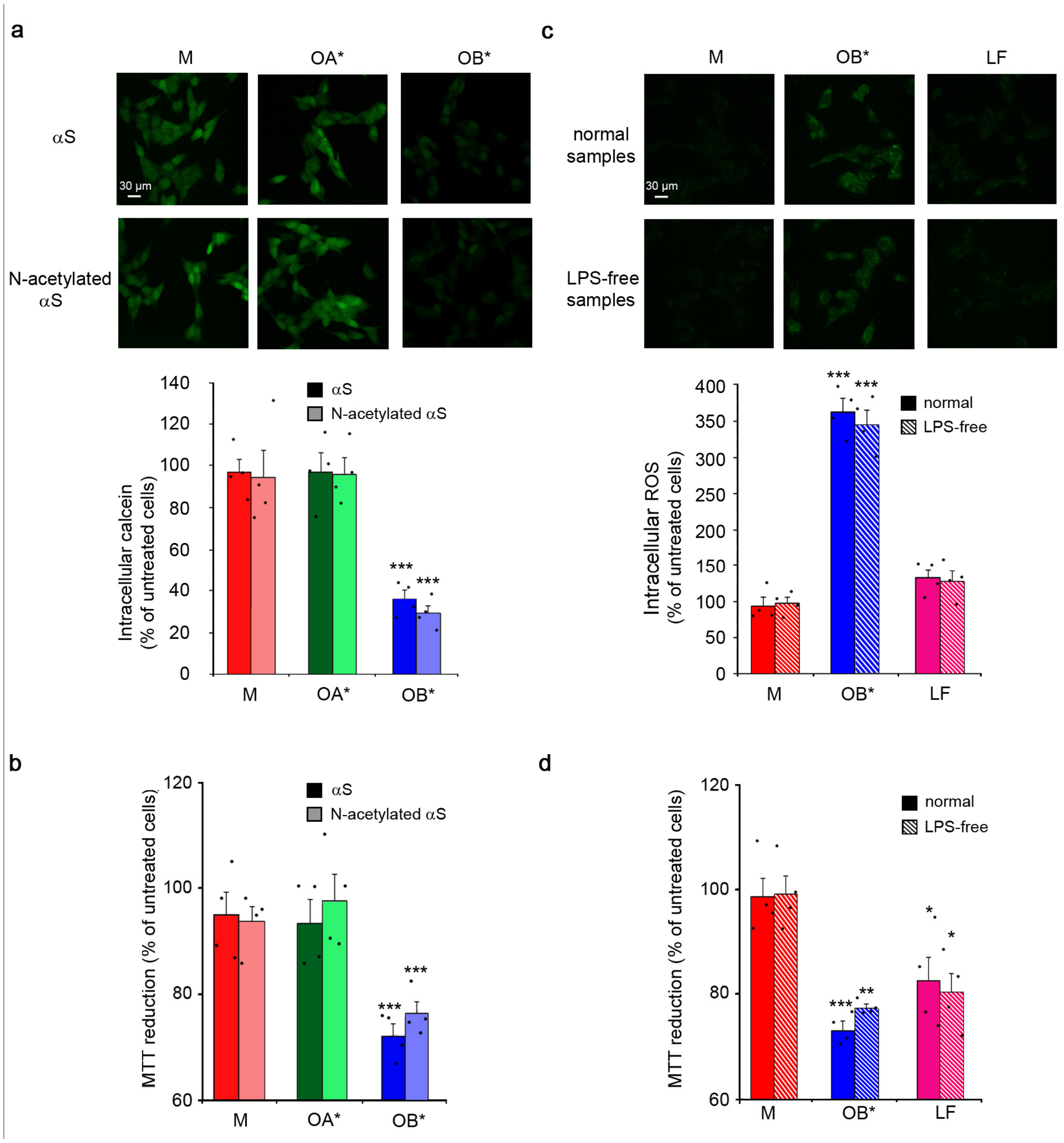

Supplementary Figure 7. Analysis of the toxicity induced by N-acetylated αS species and by LPS-free αS species (a) Representative confocal microscope images showing SH-SY5Y cells loaded with calcein-AM probe for 30 min and then treated for 1 h with the non-acetylated and N-acetylated indicated αS species (M, OA* and OB*, 0.3 μM). (**b**) MTT reduction in SH-SY5Y cells treated for 24 h with the indicated non-acetylated and N-acetylated αS species (M, OA* and OB*, 0.3 μM). **(c)** Representative confocal microscope images showing the intracellular ROS-derived fluorescence in SH-SY5Y cells treated for 15 min with normal or LPS-free αS species (M, OB* and LF, 0.3 μM). The green fluorescence arises from the CM-H_2DCFDA probe that has reacted with ROS. (**d**) MTT reduction in SH-SY5Y cells treated for 24 h with normal and LPS-free αS species (M, OB* and LF, 0.3 μM). The semi-quantitative analyses of the green fluorescence signals in A and C are expressed as the percentage of the value for untreated cells. Experimental errors are S.E.M. (n=4 with one internal replicate). In all panels, samples were analyzed by one-way ANOVA followed by Bonferroni's multiple comparison test relative to untreated cells (*P<0.05, **P<0.01, *** P<0.001). A total of 200–250 cells (**a** and **c**) and 150.000-200.000 cells (**b** and **d**) were analyzed per condition.

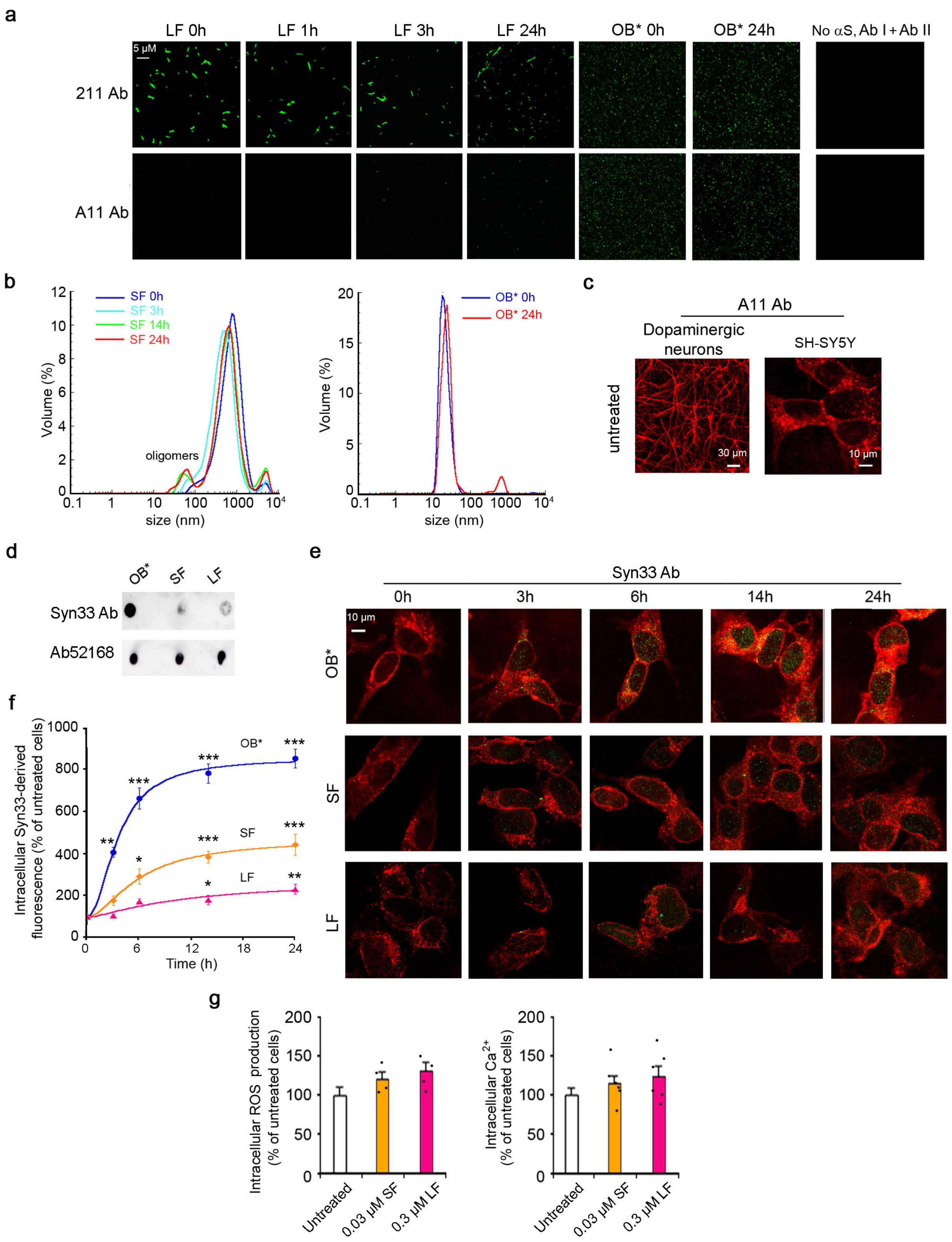

Supplementary Figure 8. αS fibrils gradually release oligomers *in vitro* and in the presence of cells. (a) Representative confocal microscope images showing 0.3 μM LF incubated in CM without cells in wells containing a glass coverslip for 0, 1, 3, 24 h at 37 °C. Representative images of OB* at 0 h and 24 h and primary and secondary antibodies without αS aggregates were also showed as positive and negative controls, respectively. The green fluorescence derives from mouse monoclonal 211 anti-αS antibodies (sc12767, Santa Cruz Biotechnology) and rabbit anti-oligomer A11 polyclonal antibodies (Thermo Fisher Scientific) and then Alexa Fluor 514-conjugated anti-mouse and anti-rabbit secondary

antibodies, respectively (three indipendent experiments with one internal replicate for human iPSC-derived dopaminergic neurons and three independent experiments with four internal replicates for SH-SY5Y cells). (**b**) Particle size distributions of SF and OB* at 1 μM in PBS following the indicated lengths of time at 37 °C. **(c)** Representative confocal scanning microscope images showing the median sections of untreated human iPSC-derived dopaminergic neurons and untreated SH-SY5Y cells. Red and green fluorescence indicates the cell membranes labeled with WGA and the A11-positive prefibrillar oligomers, respectively. **(d)** Dot-blot analysis of αS species probed with conformational specific antibodies Syn33 (ABN2265M, Sigma Aldrich) and polyclonal anti-αS antibodies (ab52168, Abcam). (**e**) Representative confocal scanning microscope images showing the median sections of SH-SY5Y cells treated for the indicated lengths of time with OB*/SF/LF at 0.3 μM. Red and green fluorescence indicates the cell membranes labeled with WGA and the Syn33-positive prefibrillar oligomers, respectively. **(f)** Kinetic plots reporting Syn33-intracellular fluorescence following the addition of 0.3 μM of the indicated αS species. The continuous lines represent the best fits to exponential and sigmoidal functions (see Methods), for OB*/SF/LF, respectively. (**g**) Semi-quantitative analyses of the intracellular ROS and Ca^{2+} -derived fluorescence in SH-SY5Y cells treated for 15 min with the indicated αS species at the indicated αS concentrations. The green fluorescence arises from the CM-H_2DCFDA and Fluo-4 AM probes, respectively. Data are expressed as the percentage of the value for untreated cells. Experimental errors are S.E.M. (in panel f n=3 with one internal replicate; in panel g n=4 with three internal replicates for ROS production and n=6 with one internal replicate for Ca^{2+} influx). In panels f and g, samples were analyzed by one-way ANOVA followed by Bonferroni's multiple comparison test relative to untreated cells (*P<0.05, **P<0.01, ***P<0.001). A total of 200–250 cells were analyzed per condition.

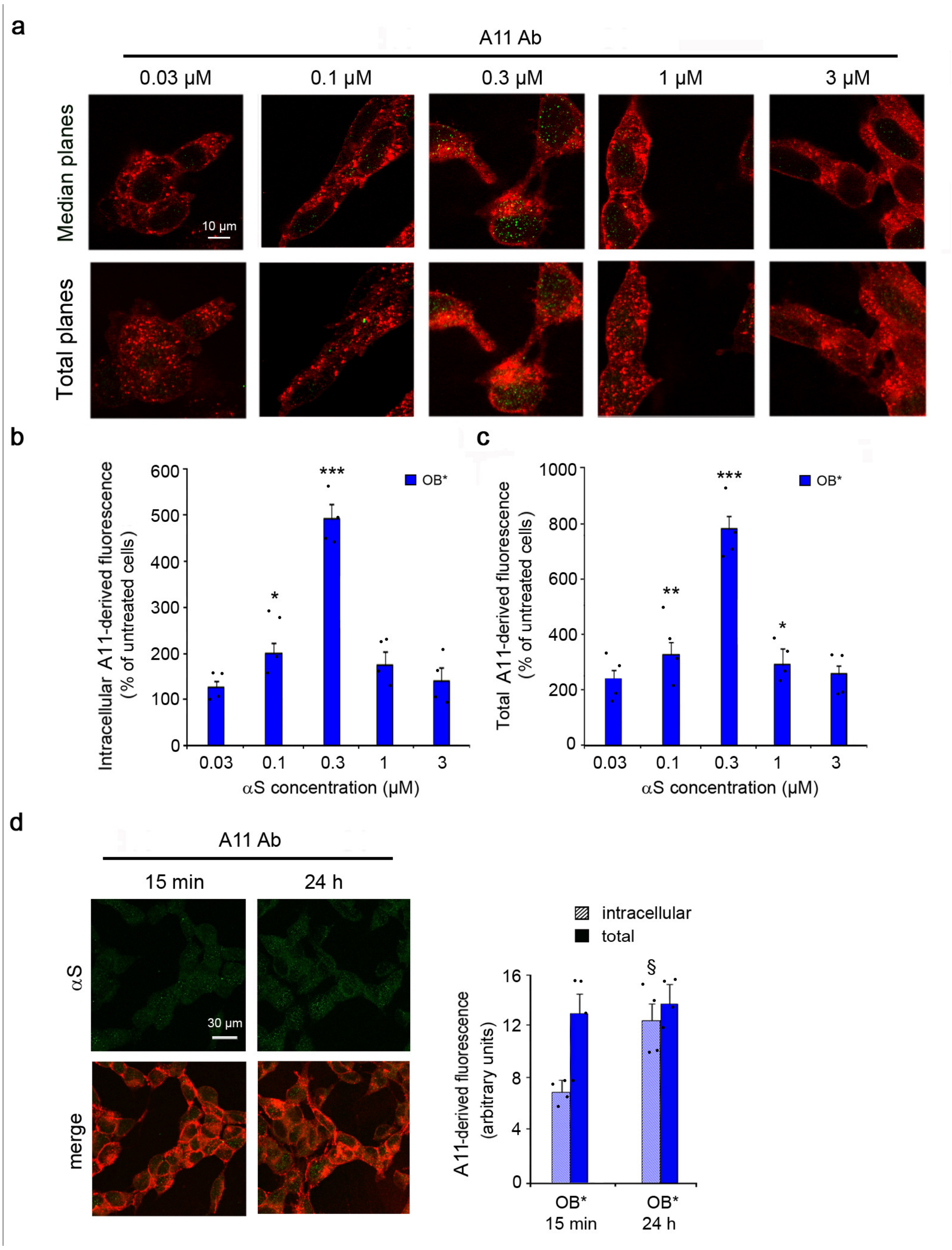

Supplementary Figure 9. Titration of OB* uptake in cells. (a) Representative confocal scanning microscope images showing the median and total sections of SH-SY5Y cells treated for 1 h with OB* at the indicated αS concentrations. Red and green fluorescence indicates the cell membranes and the αS species revealed with WGA and A11 antibodies (AHB0052, Thermo Fisher Scientific), respectively. **(b-c)** Semi-quantitative analysis of the green fluorescence signal derived from intracellular **(b)** and total **(c)** αS revealed with A11 antibodies. (**d**) Representative confocal scanning microscope images showing SH-SY5Y cells treated for 15 min and 24 h with OB* at 0.3 µM. Semi-quantitative analysis of the intracellular and total A11-derived fluorescence. Red and green fluorescence indicates the cell membranes and the αS species revealed with WGA and A11 antibodies (AHB0052, Thermo Fisher Scientific), respectively. Experimental errors are S.E.M. (n=4 with one internal replicate). In panels b-d, samples were analyzed by one-way ANOVA followed by Bonferroni's multiple comparison test relative to untreated cells (*P<0.05, **P<0.01, ***P<0.001) or to cells treated with OB* for 15 min (§P<0.05). A total of 200–250 cells were analyzed per condition.

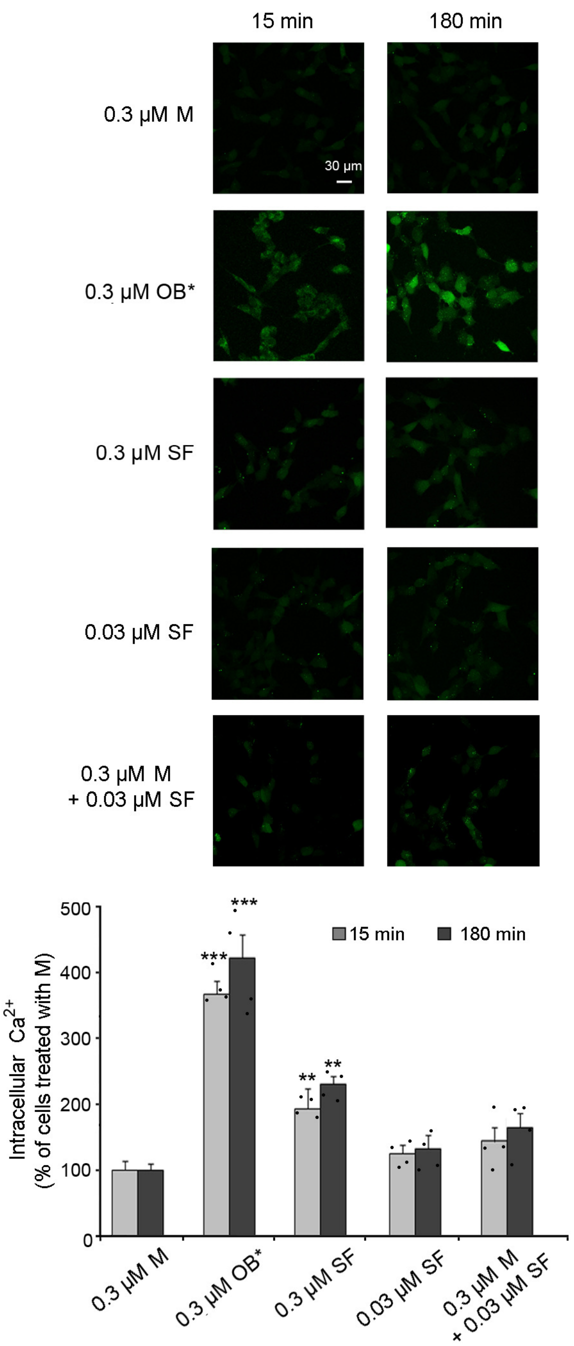

Supplementary Figure 10. Analysis of the seeding ability of αS fibrils. Representative confocal microscope images showing intracellular Ca^{2+} levels in SH-SY5Y cells treated for 15 min or 180 min with 0.3 µM M, 0.3 µM OB*, 0.3 µM SF, 0.03 µM SF and 0.3 µM M in the presence of 0.03 µM SF. Cells were loaded with Fluo-4 AM probe. Semi-quantitative analysis of the intracellular Ca^{2+}-derived fluorescence, expressed as the percentage of the value for cells treated with monomers. Experimental errors are S.E.M. (n=4 with one internal replicate). Samples were analyzed by one-way ANOVA followed by Bonferroni's multiple comparison test relative to untreated cells (**P<0.01, ***P<0.001). A total of 200–250 cells were analyzed per condition.
