# Supplementary material for: The release of toxic oligomers from α-synuclein fibrils induces dysfunction in neuronal cells
Source: Nat Commun. 2021 Mar 22;12:1814. doi: 10.1038/s41467-021-21937-3 (PMC7985515; doi:10.1038/s41467-021-21937-3)
Supplement: Supplementary file 3 — Reporting Summary [file 41467_2021_21937_MOESM3_ESM.pdf]

nature research

Corresponding author(s): N. Cremades and C. Cecchi

Last updated by author(s): Jan 29, 2021

Reporting Summary

Nature Research wishes to improve the reproducibility of the work that we publish. This form provides structure for consistency and transparency in reporting. For further information on Nature Research policies, see Authors & Referees and the Editorial Policy Checklist .

Statistics

For all statistical analyses, confirm that the following items are present in the figure legend, table legend, main text, or Methods section.

n/a	Confirmed	
☐	☒	The exact sample size (*n*) for each experimental group/condition, given as a discrete number and unit of measurement
☐	☒	A statement on whether measurements were taken from distinct samples or whether the same sample was measured repeatedly
☐	☒	The statistical test(s) used AND whether they are one- or two-sided<br>*Only common tests should be described solely by name; describe more complex techniques in the Methods section.*
☒	☐	A description of all covariates tested
☐	☒	A description of any assumptions or corrections, such as tests of normality and adjustment for multiple comparisons
☐	☒	A full description of the statistical parameters including central tendency (e.g. means) or other basic estimates (e.g. regression coefficient) AND variation (e.g. standard deviation) or associated estimates of uncertainty (e.g. confidence intervals)
☐	☒	For null hypothesis testing, the test statistic (e.g. *F*, *t*, *r*) with confidence intervals, effect sizes, degrees of freedom and *P* value noted<br>*Give P values as exact values whenever suitable.*
☒	☐	For Bayesian analysis, information on the choice of priors and Markov chain Monte Carlo settings
☒	☐	For hierarchical and complex designs, identification of the appropriate level for tests and full reporting of outcomes
☒	☐	Estimates of effect sizes (e.g. Cohen's *d*, Pearson's *r*), indicating how they were calculated

Our web collection on statistics for biologists contains articles on many of the points above.

Software and code

Policy information about availability of computer code

Data collection	AFM Images were processed with Gwyddion open source software (http://www.gwyddion.net; version 2.48); FT-IR data was performed with the Opus software package (Bruker Optics Limited, UK); X-ray diffraction data was analysed on PROTEUM 2 software suite. Dot-blot analyses were acquired using ImageQuant™ TL software (GE Healthcare UK Limited; version 8.2); confocal microscopy images were acquired using Leica Application Suite Advanced Fluorescence (LAS AF) Software (Leica Microsystems, Mannheim, Germany); STED images were acquired by using Huygens Professional software (Scientific Volume Imaging B.V., Hilversum, The Netherlands; version 18.04) and Leica Application Suite X (LAS X) software (Leica). Confocal and STED images were analyzed by the ImageJ software (National Institutes of Health, Bethesda, MD, USA; version 1.52t). MTT tests were achieved using Microplate Manager® Software (Biorad, CA, USA).
Data analysis	The statistics associated with the experiments were determined using GraphPad Prism 7.0, all using the tests as described in the methods section of the manuscript.

For manuscripts utilizing custom algorithms or software that are central to the research but not yet described in published literature, software must be made available to editors/reviewers. We strongly encourage code deposition in a community repository (e.g. GitHub). See the Nature Research guidelines for submitting code & software for further information.

Data

Policy information about availability of data

All manuscripts must include a data availability statement. This statement should provide the following information, where applicable:

- Accession codes, unique identifiers, or web links for publicly available datasets
- A list of figures that have associated raw data
- A description of any restrictions on data availability

The surce data file is avaible as supplementary file.

Field-specific reporting

Please select the one below that is the best fit for your research. If you are not sure, read the appropriate sections before making your selection.

☒ Life sciences ☐ Behavioural & social sciences ☐ Ecological, evolutionary & environmental sciences

For a reference copy of the document with all sections, see nature.com/documents/nr-reporting-summary-flat.pdf

Life sciences study design

All studies must disclose on these points even when the disclosure is negative.

Sample size	The sample size (n) used to derive statistics is correctly provided in all figure legends for each experiment
Data exclusions	No data were excluded from the study.
Replication	The reproducibility of the experimental findings was successfully verified by repeating all the experiments at least three times.
Randomization	In the relevant cell biology measurements, treatments were distributed throughout the multiwell plate using random allocation. All the biophysical measurements were performed at least in triplicate in different days using a random order of samples.
Blinding	This is an in vitro research study, thus blinding was not carried out as not relevant.

Reporting for specific materials, systems and methods

We require information from authors about some types of materials, experimental systems and methods used in many studies. Here, indicate whether each material, system or method listed is relevant to your study. If you are not sure if a list item applies to your research, read the appropriate section before selecting a response.

Materials & experimental systems

n/a	Involved in the study	
☐	☒	Antibodies
☐	☒	Eukaryotic cell lines
☒	☐	Palaeontology
☐	☒	Animals and other organisms
☒	☐	Human research participants
☒	☐	Clinical data

Methods

n/a	Involved in the study	
☒	☐	ChIP-seq
☒	☐	Flow cytometry
☒	☐	MRI-based neuroimaging

Antibodies

Antibodies used	rabbit polyclonal anti-aS antibodies (ab52168 Abcam, Cambridge, UK); rabbit anti-oligomer (A11) polyclonal antibodies (AHB0052, Thermo Fisher Scientific); rabbit anti-amyloid fibrils OC (AB2286, Sigma-Aldrich); rabbit oligomer-specific Syn33 antibody (ABN2265, Sigma-Aldrich); mouse monoclonal 211 anti-aS antibodies (sc12767, Santa Cruz Biotechnology); HRP-conjugated anti-rabbit or anti-mouse secondary antibodies (AB6721 and AB6728, Abcam); Alexa 488-conjugated anti-rabbit secondary antibody (A-11034 Thermo Fisher Scientific); Alexa-Fluor-488-conjugated anti-mouse secondary antibodies (A-11029, Thermo Fisher Scientific); Alexa Fluor 514 -goat anti mouse IgG1 secondary antibody (A-31555 Thermo Fisher Scientific); Alexa-Fluor-568-conjugated goat anti-rabbit secondary antibodies (A-11036 Thermo Fisher Scientific); rabbit anti-MAP2 antibodies (ab32454, Abcam); mouse anti-TH antibodies (sc-25269, Santa Cruz Biotechnology); mouse anti-MAP2 antibodies (ab11267, Abcam).
Validation	Rabbit polyclonal anti-aS antibodies (ab52168 Abcam, Cambridge, UK)<br>Specificity: Due to 83% sequence homology ab52168 might react with Beta synuclein<br>Suitable for: IHC-P<br>Species reactivity Reacts with: Mouse, Rat, Human<br>Immunogen: Synthetic peptide corresponding to Human Alpha-synuclein. Synthetic non-phosphopeptide<br>derived from human alpha Synuclein around the phosphorylation site of tyrosine 133 (E-G-Y-Q-D).<br><br>Anti-oligomer (A11) polyclonal antibodies (AHB0052, Thermo Fisher Scientific)<br>This antibody recognizes amino acid sequence-independent oligomers of proteins or peptides. A11 does not recognize monomers or mature fibers of proteins or peptides. A11 reacts with soluble AB40 oligomers and does not react with soluble low molecular weight AB40 or AB40 fibrils. A11 recognizes oligomeric species of several other amyloidogenic polypeptides including AB42, human insulin, prion, polyglutamine, lysozyme, alpha-synuclein and yeast prion Sup35.<br>Rabbit anti-amyloid fibrils OC (AB2286, Sigma-Aldrich)

This antibody recognizes generic epitopes common to many amyloid fibrils and fibrillar oligomers, but not prefibrillar oligomers or natively folded proteins. It may also show weak reactivity against Aβ monomers while AB2287 does not.

Rabbit oligomer-specific Syn33 antibody (ABN2265, Sigma-Aldrich)
Specificity This rabbit polyclonal antibody specifically recognizes aggregated form of alpha-synuclein.
Immunogen Synuclein oligomers from full length human wild-type alpha-synuclein.

mouse monoclonal 211 anti-aS antibodies (sc12767, Santa Cruz Biotechnology)
α-synuclein (211) is a mouse monoclonal antibody raised against amino acids 121-125 of α-synuclein of human origin.
α-synuclein (211) is recommended for detection of α-synuclein of human origin by Western Blotting (starting dilution 1:200, dilution range 1:100-1:1000), immunoprecipitation [1-2 µg per 100-500 µg of total protein (1 ml of cell lysate)], immunofluorescence (starting dilution 1:50, dilution range 1:50-1:500) and immunohistochemistry (including paraffin-embedded sections) (starting dilution 1:50, dilution range 1:50-1:500). Suitable for use as control antibody for α-synuclein siRNA (h): sc-29619, α-synuclein shRNA Plasmid (h): sc-29619-SH and α-synuclein shRNA (h) Lentiviral Particles: sc-29619-V.

Eukaryotic cell lines

Policy information about cell lines

Cell line source(s)	SH-SY5Y human neuroblastoma cells were acquired from A.T.C.C. (VA, USA). Primary cortical neurons were obtained from embryonic day (ED)-17 Sprague-Dawley rats (Harlan). iPSC-derived dopaminergic neurons were obtained from Axol Bioscience (Cambridge, UK)
Authentication	SH-SY5Y cell line was authenticated by the European Collection of Authenticated Cell Cultures using short tandem repeat loci analyses. Primary cortical neurons were authenticated using PSD-95 as mature neuronal marker. iPSC-derived dopaminergic neurons were uthenticated using tyrosine-hydroxylase as mature neuronal marker
Mycoplasma contamination	The cell lines were tested negative for mycoplasma contaminations.
Commonly misidentified lines (See ICLAC register)	No commonly misidentified cell lines were used in this study.

Animals and other organisms

Policy information about studies involving animals; ARRIVE guidelines recommended for reporting animal research

Laboratory animals	6-month old Sprague-Dawley rats (Harlan), timed pregnant females.
Wild animals	No wild animals were used in this study.
Field-collected samples	No field collected samples were used in this study.
Ethics oversight	As described in the methods section of the manuscript, experiments and animal use procedures were in accordance with the National Institutes of Health Guide for the Care and Use of Laboratory Animals. The experimental protocols were approved by the "Commissione per l'Etica della ricerca" of the University of Florence, in compliance with the European Convention for the Protection of Vertebrate Animals used for Experimental and Other Scientific Purposes and the European Communities Council. The authors further attest that all efforts were made to minimize the number of animals used and their suffering.

Note that full information on the approval of the study protocol must also be provided in the manuscript.
